# 1-(2,3-Di­cyano­phen­yl)pyridin-1-ium-4-olate monohydrate

**DOI:** 10.1107/S1600536813008891

**Published:** 2013-04-20

**Authors:** Zhe Han, Ying Zhu, Xuelei Tian, Suping Feng

**Affiliations:** a School of Materials Science and Engineering of Shandong University, 250014 Jinan, People’s Republic of China; b New Materials Institute of Shandong Academy of Sciences, 250014 Jinan, People’s Republic of China; c School of Environmental Science and Engineering of Shandong University, 250100 Jinan, People’s Republic of China

## Abstract

In the crystal structure of the title compound, C_13_H_7_N_3_O·H_2_O, the components are associated into chains along [010] through strong O—H⋯O hydrogen bonds with the free water mol­ecules as bridging ligands. These chains are further cross-linked by C—H⋯O and C—H⋯N hydrogen bonds, forming a three-dimensional structure.

## Related literature

For the preparation of the title compound, see: Archibald *et al.* (1994 ▶)
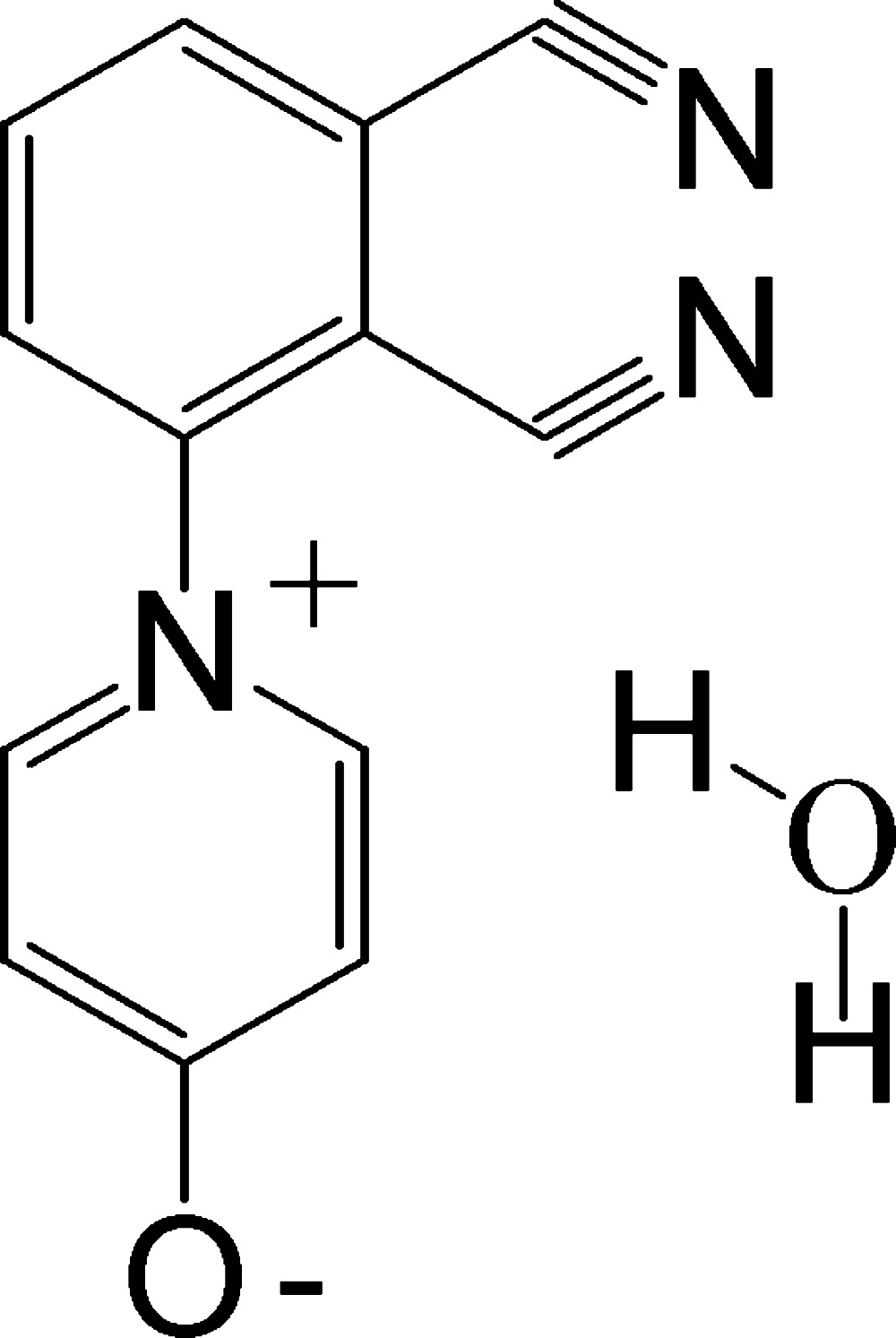


## Experimental

### 

#### Crystal data

C_13_H_7_N_3_O·H_2_O
*M* *_r_* = 239.23
Monoclinic, 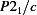

*a* = 11.977 (2) Å
*b* = 7.2497 (12) Å
*c* = 13.850 (2) Å
β = 94.244 (3)°
*V* = 1199.3 (3) Å^3^
*Z* = 4
Mo *K*α radiation
μ = 0.09 mm^−1^
*T* = 293 K
0.12 × 0.10 × 0.08 mm

#### Data collection

Bruker APEXII CCD diffractometer
Absorption correction: multi-scan (*SADABS*; Bruker, 2001 ▶) *T*_min_ = 0.989, *T*_max_ = 0.993
5795 measured reflections
2112 independent reflections
1349 reflections with *I* > 2σ(*I*)
*R*_int_ = 0.042

#### Refinement

*R*[*F* ^2^ > 2σ(*F* ^2^)] = 0.044
*wR*(*F* ^2^) = 0.124
*S* = 1.03
2112 reflections
172 parameters
H atoms treated by a mixture of independent and constrained refinement
Δρ_max_ = 0.12 e Å^−3^
Δρ_min_ = −0.14 e Å^−3^

### 

Data collection: *APEX2* (Bruker, 2004 ▶); cell refinement: *SAINT-Plus* (Bruker, 2001 ▶); data reduction: *SAINT-Plus*; program(s) used to solve structure: *SHELXS97* (Sheldrick, 2008 ▶); program(s) used to refine structure: *SHELXL97* (Sheldrick, 2008 ▶); molecular graphics: *SHELXTL* (Sheldrick, 2008 ▶); software used to prepare material for publication: *SHELXTL*.

## Supplementary Material

Crystal structure: contains datablock(s) global, I. DOI: 10.1107/S1600536813008891/bv2216sup1.cif

Structure factors: contains datablock(s) I. DOI: 10.1107/S1600536813008891/bv2216Isup2.hkl

Supplementary material file. DOI: 10.1107/S1600536813008891/bv2216Isup3.cml

Additional supplementary materials:  crystallographic information; 3D view; checkCIF report

## Figures and Tables

**Table 1:** Hydrogen-bond geometry (Å, °)

*D*—H⋯*A*	*D*—H	H⋯*A*	*D*⋯*A*	*D*—H⋯*A*
O1*W*—H1*WA*⋯O1	0.98 (3)	1.79 (3)	2.763 (2)	170 (3)
O1*W*—H1*WB*⋯O1^i^	0.87 (3)	1.88 (3)	2.721 (2)	162 (3)
C6—H6*A*⋯O1*W* ^ii^	0.93	2.37	3.302 (3)	177
C13—H13*A*⋯O1*W* ^iii^	0.93	2.38	3.311 (3)	175
C9—H9*A*⋯N1^iv^	0.93	2.55	3.440 (2)	160

Symmetry codes: (i) 
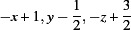
; (ii) 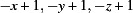; (iii) 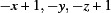; (iv) 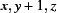.

**Fig. 1.**
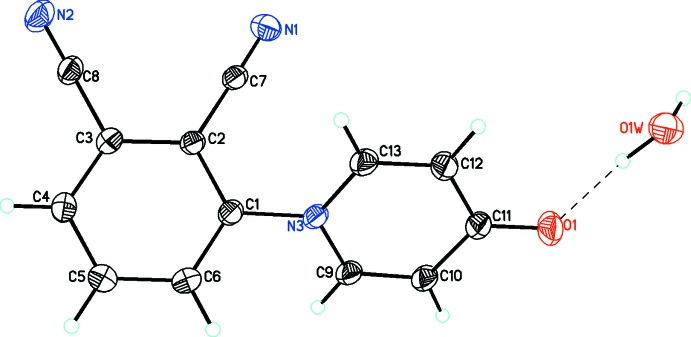
The asymmetric unit of the title compound with the atom-labelling scheme. Displacement ellipsoids are drawn at the 30% probability level; H atoms are given as spheres of arbitrary radius.

**Fig. 2.**
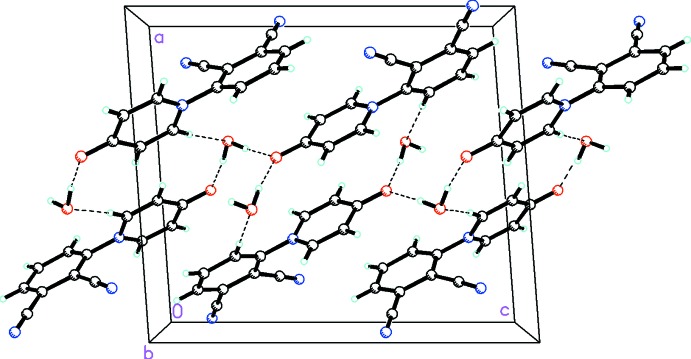
The packing diagram of the title compound with hydrogen bonds shown as dashed lines.

## supplementary crystallographic information

### Comment

In the crystal structure of the title compound, (C_13_H_7_N_3_O)(H_2_O), the units are associated into a chain through strong O—H···O hydrogen bonds with the free water molecules as the bridging ligands. These chains are further crosslinked by C-H···O interactions. In addition there are weak pairwise C—H···N hydrogen bonds.

### Experimental

The preparation of the title compound, see, Archibald *et al.* (1994)

### Refinement

All hydrogen atoms bound to carbon were refined using a riding model with distance C—H = 0.93 Å, *U*_iso_ = 1.2*U*_eq_ (C) for aromatic atoms. The H atoms of the water molecule were located from difference density maps and were refined isotrpically.

### Crystal data

**Table:**

C_13_H_7_N_3_O·H_2_O	*F*(000) = 496
*M**_r_* = 239.23	*D*_x_ = 1.325 Mg m^−^^3^
Monoclinic, *P*2_1_/*c*	Mo *K*α radiation, λ = 0.71073 Å
Hall symbol: -P 2ybc	Cell parameters from 2117 reflections
*a* = 11.977 (2) Å	θ = 1.7–25.0°
*b* = 7.2497 (12) Å	µ = 0.09 mm^−^^1^
*c* = 13.850 (2) Å	*T* = 293 K
β = 94.244 (3)°	Block, colorless
*V* = 1199.3 (3) Å^3^	0.12 × 0.10 × 0.08 mm
*Z* = 4	

### Data collection

**Table:**

Bruker APEXII CCD diffractometer	2112 independent reflections
Radiation source: fine-focus sealed tube	1349 reflections with *I* > 2σ(*I*)
Graphite monochromator	*R*_int_ = 0.042
φ and ω scans	θ_max_ = 25.0°, θ_min_ = 1.7°
Absorption correction: multi-scan (*SADABS*; Bruker, 2001)	*h* = −14→14
*T*_min_ = 0.989, *T*_max_ = 0.993	*k* = 0→8
5795 measured reflections	*l* = 0→16

### Refinement

**Table:**

Refinement on *F*^2^	Secondary atom site location: difference Fourier map
Least-squares matrix: full	Hydrogen site location: inferred from neighbouring sites
*R*[*F*^2^ > 2σ(*F*^2^)] = 0.044	H atoms treated by a mixture of independent and constrained refinement
*wR*(*F*^2^) = 0.124	*w* = 1/[σ^2^(*F*_o_^2^) + (0.0572*P*)^2^] where *P* = (*F*_o_^2^ + 2*F*_c_^2^)/3
*S* = 1.03	(Δ/σ)_max_ < 0.001
2112 reflections	Δρ_max_ = 0.12 e Å^−^^3^
172 parameters	Δρ_min_ = −0.14 e Å^−^^3^
0 restraints	Extinction correction: *SHELXL97* (Sheldrick, 2008), Fc^*^=kFc[1+0.001xFc^2^λ^3^/sin(2θ)]^-1/4^
Primary atom site location: structure-invariant direct methods	Extinction coefficient: 0.015 (4)

### Special details

**Table:**

Geometry. All esds (except the esd in the dihedral angle between two l.s. planes) are estimated using the full covariance matrix. The cell esds are taken into account individually in the estimation of esds in distances, angles and torsion angles; correlations between esds in cell parameters are only used when they are defined by crystal symmetry. An approximate (isotropic) treatment of cell esds is used for estimating esds involving l.s. planes.
Refinement. Refinement of F^2^ against ALL reflections. The weighted R-factor wR and goodness of fit S are based on F^2^, conventional R-factors R are based on F, with F set to zero for negative F^2^. The threshold expression of F^2^ > 2sigma(F^2^) is used only for calculating R-factors(gt) etc. and is not relevant to the choice of reflections for refinement. R-factors based on F^2^ are statistically about twice as large as those based on F, and R- factors based on ALL data will be even larger.

### Fractional atomic coordinates and isotropic or equivalent isotropic displacement parameters (Å^2^)

**Table:**

	*x*	*y*	*z*	*U*_iso_*/*U*_eq_	
O1	0.44687 (15)	0.4052 (2)	0.64729 (11)	0.0821 (6)	
N1	0.14211 (16)	−0.0800 (2)	0.38040 (14)	0.0686 (6)	
N2	0.00645 (18)	−0.1230 (3)	0.11391 (13)	0.0794 (7)	
N3	0.29064 (13)	0.35472 (19)	0.37786 (11)	0.0477 (5)	
C1	0.23465 (16)	0.3377 (2)	0.28309 (13)	0.0469 (5)	
C2	0.17290 (15)	0.1798 (2)	0.25806 (13)	0.0437 (5)	
C3	0.11900 (16)	0.1688 (2)	0.16464 (14)	0.0479 (5)	
C4	0.12482 (18)	0.3119 (3)	0.09995 (14)	0.0578 (6)	
H4A	0.0889	0.3032	0.0383	0.069*	
C5	0.1844 (2)	0.4680 (3)	0.12749 (15)	0.0652 (7)	
H5A	0.1875	0.5659	0.0844	0.078*	
C6	0.23959 (19)	0.4810 (3)	0.21802 (16)	0.0609 (6)	
H6A	0.2803	0.5867	0.2354	0.073*	
C7	0.15735 (17)	0.0352 (3)	0.32680 (15)	0.0482 (5)	
C8	0.05625 (18)	0.0052 (3)	0.13666 (14)	0.0561 (6)	
C9	0.27488 (17)	0.5072 (2)	0.43253 (15)	0.0536 (6)	
H9A	0.2273	0.5997	0.4075	0.064*	
C10	0.32575 (18)	0.5283 (3)	0.52132 (15)	0.0567 (6)	
H10A	0.3128	0.6349	0.5561	0.068*	
C11	0.39919 (18)	0.3909 (3)	0.56354 (15)	0.0578 (6)	
C12	0.41474 (18)	0.2360 (3)	0.50307 (15)	0.0618 (6)	
H12A	0.4627	0.1421	0.5257	0.074*	
C13	0.36240 (17)	0.2209 (3)	0.41422 (14)	0.0570 (6)	
H13A	0.3752	0.1176	0.3768	0.068*	
O1W	0.60702 (15)	0.1549 (2)	0.71708 (14)	0.0732 (5)	
H1WA	0.544 (2)	0.236 (4)	0.6961 (19)	0.121 (11)*	
H1WB	0.583 (2)	0.095 (4)	0.766 (2)	0.120 (12)*	

### Atomic displacement parameters (Å^2^)

**Table:**

	*U*^11^	*U*^22^	*U*^33^	*U*^12^	*U*^13^	*U*^23^
O1	0.1059 (14)	0.0738 (11)	0.0615 (10)	0.0203 (9)	−0.0291 (9)	−0.0165 (8)
N1	0.0756 (14)	0.0545 (11)	0.0750 (14)	−0.0007 (9)	0.0007 (11)	0.0099 (10)
N2	0.0927 (16)	0.0754 (13)	0.0685 (14)	−0.0333 (12)	−0.0050 (11)	−0.0101 (10)
N3	0.0482 (10)	0.0384 (9)	0.0549 (10)	0.0008 (7)	−0.0071 (8)	−0.0087 (7)
C1	0.0472 (12)	0.0433 (11)	0.0494 (12)	−0.0008 (9)	−0.0022 (9)	−0.0062 (9)
C2	0.0458 (12)	0.0385 (10)	0.0467 (11)	−0.0013 (8)	0.0019 (9)	−0.0029 (8)
C3	0.0481 (12)	0.0464 (11)	0.0492 (12)	−0.0050 (9)	0.0033 (9)	−0.0060 (9)
C4	0.0663 (15)	0.0606 (13)	0.0452 (12)	−0.0070 (11)	−0.0046 (10)	−0.0017 (10)
C5	0.0849 (17)	0.0540 (13)	0.0557 (14)	−0.0102 (12)	−0.0026 (12)	0.0070 (10)
C6	0.0718 (16)	0.0459 (12)	0.0637 (15)	−0.0143 (11)	−0.0042 (12)	−0.0005 (10)
C7	0.0487 (13)	0.0419 (11)	0.0531 (13)	−0.0006 (9)	−0.0016 (10)	−0.0078 (10)
C8	0.0590 (14)	0.0586 (13)	0.0497 (13)	−0.0132 (11)	−0.0019 (10)	−0.0053 (10)
C9	0.0571 (14)	0.0377 (11)	0.0640 (14)	0.0032 (9)	−0.0082 (11)	−0.0077 (9)
C10	0.0642 (14)	0.0435 (11)	0.0608 (14)	0.0065 (10)	−0.0062 (11)	−0.0141 (9)
C11	0.0654 (15)	0.0504 (12)	0.0555 (14)	0.0030 (11)	−0.0088 (11)	−0.0090 (10)
C12	0.0653 (16)	0.0503 (12)	0.0667 (15)	0.0159 (10)	−0.0148 (11)	−0.0088 (10)
C13	0.0585 (14)	0.0434 (11)	0.0673 (14)	0.0088 (10)	−0.0082 (11)	−0.0128 (10)
O1W	0.0750 (13)	0.0633 (11)	0.0813 (13)	0.0009 (9)	0.0063 (10)	0.0050 (9)

### Geometric parameters (Å, º)

**Table:**

O1—C11	1.259 (2)	C5—C6	1.376 (3)
N1—C7	1.141 (2)	C5—H5A	0.9300
N2—C8	1.136 (2)	C6—H6A	0.9300
N3—C9	1.361 (2)	C9—C10	1.340 (3)
N3—C13	1.367 (2)	C9—H9A	0.9300
N3—C1	1.434 (2)	C10—C11	1.425 (3)
C1—C6	1.379 (3)	C10—H10A	0.9300
C1—C2	1.393 (2)	C11—C12	1.421 (3)
C2—C3	1.405 (2)	C12—C13	1.343 (2)
C2—C7	1.437 (3)	C12—H12A	0.9300
C3—C4	1.375 (3)	C13—H13A	0.9300
C3—C8	1.442 (3)	O1W—H1WA	0.98 (3)
C4—C5	1.377 (3)	O1W—H1WB	0.87 (3)
C4—H4A	0.9300		
			
C9—N3—C13	118.73 (16)	C1—C6—H6A	119.9
C9—N3—C1	120.28 (15)	N1—C7—C2	178.2 (2)
C13—N3—C1	120.98 (15)	N2—C8—C3	179.4 (2)
C6—C1—C2	120.27 (17)	C10—C9—N3	122.07 (18)
C6—C1—N3	119.57 (16)	C10—C9—H9A	119.0
C2—C1—N3	120.14 (16)	N3—C9—H9A	119.0
C1—C2—C3	118.35 (16)	C9—C10—C11	121.43 (18)
C1—C2—C7	121.80 (16)	C9—C10—H10A	119.3
C3—C2—C7	119.73 (16)	C11—C10—H10A	119.3
C4—C3—C2	121.03 (17)	O1—C11—C12	122.52 (18)
C4—C3—C8	119.71 (17)	O1—C11—C10	123.01 (18)
C2—C3—C8	119.26 (17)	C12—C11—C10	114.47 (18)
C3—C4—C5	119.31 (18)	C13—C12—C11	122.10 (18)
C3—C4—H4A	120.3	C13—C12—H12A	119.0
C5—C4—H4A	120.3	C11—C12—H12A	119.0
C6—C5—C4	120.84 (19)	C12—C13—N3	121.17 (17)
C6—C5—H5A	119.6	C12—C13—H13A	119.4
C4—C5—H5A	119.6	N3—C13—H13A	119.4
C5—C6—C1	120.18 (18)	H1WA—O1W—H1WB	104 (3)
C5—C6—H6A	119.9		
			
C9—N3—C1—C6	50.0 (3)	C2—C1—C6—C5	−0.7 (3)
C13—N3—C1—C6	−128.6 (2)	N3—C1—C6—C5	−178.7 (2)
C9—N3—C1—C2	−128.0 (2)	C1—C2—C7—N1	140 (7)
C13—N3—C1—C2	53.4 (3)	C3—C2—C7—N1	−36 (7)
C6—C1—C2—C3	1.7 (3)	C4—C3—C8—N2	−9 (28)
N3—C1—C2—C3	179.77 (17)	C2—C3—C8—N2	171 (100)
C6—C1—C2—C7	−174.18 (19)	C13—N3—C9—C10	−1.3 (3)
N3—C1—C2—C7	3.9 (3)	C1—N3—C9—C10	−179.9 (2)
C1—C2—C3—C4	−1.4 (3)	N3—C9—C10—C11	−0.2 (3)
C7—C2—C3—C4	174.61 (19)	C9—C10—C11—O1	−178.9 (2)
C1—C2—C3—C8	178.93 (18)	C9—C10—C11—C12	1.4 (3)
C7—C2—C3—C8	−5.1 (3)	O1—C11—C12—C13	179.2 (2)
C2—C3—C4—C5	0.0 (3)	C10—C11—C12—C13	−1.1 (3)
C8—C3—C4—C5	179.7 (2)	C11—C12—C13—N3	−0.4 (3)
C3—C4—C5—C6	1.1 (3)	C9—N3—C13—C12	1.6 (3)
C4—C5—C6—C1	−0.8 (3)	C1—N3—C13—C12	−179.8 (2)

### Hydrogen-bond geometry (Å, º)

**Table:**

*D*—H···*A*	*D*—H	H···*A*	*D*···*A*	*D*—H···*A*
O1*W*—H1*WA*···O1	0.98 (3)	1.79 (3)	2.763 (2)	170 (3)
O1*W*—H1*WB*···O1^i^	0.87 (3)	1.88 (3)	2.721 (2)	162 (3)
C6—H6*A*···O1*W*^ii^	0.93	2.37	3.302 (3)	177
C13—H13*A*···O1*W*^iii^	0.93	2.38	3.311 (3)	175
C9—H9*A*···N1^iv^	0.93	2.55	3.440 (2)	160

Symmetry codes: (i) −*x*+1, *y*−1/2, −*z*+3/2; (ii) −*x*+1, −*y*+1, −*z*+1; (iii) −*x*+1, −*y*, −*z*+1; (iv) *x*, *y*+1, *z*.
